# Mitral annular plane systolic excursion for assessing left ventricular systolic dysfunction in patients with septic shock

**DOI:** 10.1016/j.bjao.2023.100220

**Published:** 2023-08-12

**Authors:** Clément Brault, Yoann Zerbib, Pablo Mercado, Momar Diouf, Audrey Michaud, Christophe Tribouilloy, Julien Maizel, Michel Slama

**Affiliations:** 1 Intensive Care Department, Amiens-Picardie University Hospital, Amiens, France; 2 Universidad del Desarrollo, Departamento de Paciente Crítico, Facultad de Medicina Clínica Alemana, Santiago, Chile; 3 Clinical Research Department Amiens-Picardie University Hospital, Amiens, France; 4 Department of Cardiology, Amiens-Picardie University Hospital, Amiens, France

**Keywords:** global longitudinal strain, left ventricular ejection fraction, mitral annular plane systolic excursion, septic cardiomyopathy, septic shock

## Abstract

**Background:**

Using easy-to-determine bedside measurements, we developed an echocardiographic algorithm for predicting left ventricular ejection fraction (LVEF) and longitudinal strain (LVLS) in patients with septic shock.

**Methods:**

We measured septal and lateral mitral annular plane systolic excursion (MAPSE), septal and lateral mitral S-wave velocity, and the left ventricular longitudinal wall fractional shortening in patients with septic shock. We used a conditional inference tree method to build a stratification algorithm. The left ventricular systolic dysfunction was defined as an LVEF <50%, an LVLS greater than −17%, or both.

**Results:**

We included 71 patients (males: 61%; mean [standard deviation] age: 61 [15] yr). Septal MAPSE (cut-off: 1.2 cm) was the best predictor of left ventricular systolic dysfunction. The level of agreement between the septal MAPSE and the left ventricular systolic dysfunction was 0.525 [0.299–0.751]. A septal MAPSE ≥1.2 cm predicted normal LVEF in 17/18 patients, or 94%. In contrast, a septal MAPSE <1.2 cm predicted left ventricular systolic dysfunction with impaired LVLS in 46/53 patients (87%), although 32/53 (60%) patients had a preserved LVEF.

**Conclusions:**

Septal MAPSE is easily measured at the bedside and might help clinicians to detect left ventricular systolic dysfunction early—especially when myocardial strain measurements are not feasible.

Bedside transthoracic echocardiography (TTE) is the cornerstone of the diagnosis and management of left ventricular (LV) systolic dysfunction in critically ill patients, especially those with septic shock.^1^^,^^2^ Indeed, the incidence rate of cardiac dysfunction during sepsis (also referred to as septic cardiomyopathy [SCM]) ranges from 14% to 40%, depending on the diagnostic criteria used.^3^ Because of the absence of a consensus definition, the diagnosis and the management of SCM are challenging for clinicians. The impact of SCM on mortality is still subject to debate and might depend on the method used to assess overall LV performance.^4^

At present, the estimated left ventricular ejection fraction (LVEF) is the primary marker of LV systolic function in critically ill patients but it has several limitations.^5^ Firstly, it may be difficult to measure LVEF accurately in patients with tachycardia, mild-to-moderate systolic dysfunction, or low echogenicity. Secondly, LVEF is sensitive to the loading conditions (i.e. preload and afterload) and does not directly reflect intrinsic LV contractility.^6^ These limitations might explain why LVEF was found not to predict mortality in patients with SCM.^6^, ^7^, ^8^, ^9^, ^10^, ^11^

Myocardial strain (as measured using two-dimensional [2D] speckle-tracking echocardiography) might facilitate the early diagnosis of SCM by detecting early LV systolic dysfunction (i.e. before a decrease in LVEF). A recent meta-analysis by Sanfilippo and colleagues^8^ showed that global longitudinal strain (GLS) was strongly associated with mortality (standard mean difference −0.26; *P*=0.02). Experts have prompted clinicians to report markers of systolic function other than LVEF and GLS, such as the mitral annular plane systolic excursion (MAPSE) and the systolic myocardial velocity (S-wave) at the mitral annulus.^12^ These variables might be less load-dependent than LVEF and more closely related to LV contractility. Recently, Huang and colleagues^13^ developed the measurement of LV longitudinal wall fractional shortening (LV-LWFS) as a new surrogate marker for LV systolic function. LV-LWFS is calculated from the MAPSE and the LV length, and therefore requires a standard ultrasound device only. Although Johansson Blixt and colleagues^14^ observed a strong correlation between LV-LWFS and left ventricular longitudinal strain (LVLS) (especially in patients with septic shock), there are few published data concerning the correlation of LV-LWFS with LVEF.

The objective of the present study was to use echocardiographic variables (i.e. MAPSE, the mitral S-wave velocity, and LV-LWFS) that are easily and reproducibly measured at the bedside to develop an algorithm for predicting LV systolic dysfunction (defined as an LVEF <50%, an LVLS greater than −17%, or both) in patients with septic shock.

## Methods

### Study design

This prospective study was conducted in the intensive care unit (ICU) at Amiens University Hospital (Amiens, France). We included all consecutive patients on mechanical ventilation and admitted with septic shock or having developed septic shock during their stay in the ICU between March 2015 and January 2017. Septic shock was defined according to the Sepsis-2 criteria.^15^ The exclusion criteria were as follows: arrhythmia, severe mitral or aortic valvulopathy, prosthetic valves, or inadequate image quality for Doppler measurements. The septic shock was treated in accordance with international guidelines. Noradrenaline was the first-choice vasopressor for targeting a mean arterial pressure of 65 mm Hg. Cardiac function was evaluated with a comprehensive TTE examination during the first 72 h of septic shock. None of the patients received dobutamine before TTE.

The study protocol was approved by an independent ethics committee (CPP Nord-Ouest II, Amiens, France; reference: CEERNI 110) and registered with the French National Data Protection Commission (*Commission Nationale de l'Informatique et des Libertés*, Paris, France: registration number 2208336). Informed consent was obtained from the patient's family or (after recovery) the patient.

### Echocardiographic assessments

Comprehensive 2D and Doppler echocardiography assessments were performed using a VIVID (Vivid S6) system with a 1.5–4.5 MHz transducer (GE Medical Systems, Milwaukee, WI, USA). The depth and sector angle were adjusted to capture the entire left ventricle, including the septal and lateral mitral annuli. All measurements complied with the European Association of Cardiovascular Imaging/American Society of Echocardiography guidelines and were averaged over five consecutive cardiac cycles.^5^^,^^16^ All measurements were performed independently by investigators with expertise in critical care echocardiography (PM and MS). The PRICES checklist is available in Supplementary Table S1.

Based on the LV end-diastolic and end-systolic volumes, we calculated the LVEF (in %) according to Simpson's modified method.^17^ Using an offline speckle-tracking analysis in EchoPAC software (version 203, GE Vingmed Ultrasound AS, Horten, Norway), we assessed the LVLS (%).^18^ Briefly, we identified three points on a diastolic frame—the septal and lateral mitral annulus and the apex of the left ventricle—in the apical four-chamber view. The software automatically detected the endocardial border and traced the region of interest (ROI). The ROI was adjusted to exclude the pericardium, trabeculae, and papillary muscles. Next, the software automatically divided the LV wall into six segments and generated time-segment strain curves for a single cardiac cycle. The LVLS was defined as the average longitudinal strain at end-systole in the four-apical chamber view. The mean frame rate in the speckle-tracking strain assessment was 56 s^−1^. In the present study, LV systolic dysfunction was defined as an LVEF <50%, an LVLS greater than −17% (i.e. less negative than −17%), or both.^19^^,^^20^

Using the four-apical chamber view, we measured the following TTE variables as surrogate markers of LV systolic function. We then built a stratification algorithm for predicting LVEF and LVLS, as explained below:

1. MAPSE: in conventional M-mode, we measured the longitudinal excursion of the septal and lateral mitral annuli. Given that M-mode is angle dependent, we made sure that the M-mode line was parallel to the LV walls and that the deviation was below 15 degrees. MAPSE (cm) was measured as the distance between the peak (i.e. end-systole) and bottom (i.e. end-diastole) of the M-mode tracing curve.^21^
2. Mitral S-wave: we placed the sample volume in the ventricular myocardium, immediately adjacent to the mitral annulus. We then used pulsed-wave tissue Doppler imaging to measure the peak systolic velocity (S-wave, in cm s^−1^) in both septal and lateral mitral annuli.^22^
3. LV-LWFS: using the method by Huang and colleagues,^13^ we calculated the LV-LWFS (%) as the ratio between the total MAPSE (i.e. the sum of the septal and lateral MAPSE) and the total M-mode LV length (i.e. the sum of the septal and lateral LV lengths).

## Statistics

Data were expressed as the mean (standard deviation [SD]), median (inter-quartile range [IQR]), or frequency (%), as appropriate. Spearman's correlation coefficient was used to analyse the correlation between LV systolic function as estimated by different methods. Correlation analysis (Pearson's correlation coefficient) and Bland–Altman plots were used to assess intra- and inter-observer reliabilities of echocardiographic variables in a sample of 20 patients. Next, we used a conditional inference tree method to build our stratification algorithm; recursive partitioning was used to stratify the population of interest into subgroups.^23^ The best predictor and the corresponding optimal cut-off value (the value that splits the current node into two child nodes, with the greatest difference in the response variable) were calculated. To limit the impact of outliers, all input predictors were log-transformed. Multiple imputation with three replications was performed on the independent variables before the tree building process so that all study patients were considered (distribution of the missing variables is shown in Supplementary Table S2). Bonferroni's correction for multiple testing was applied to univariate *P*-values. The tree-building process continued as long as a predictor led to significantly different child nodes. The final sets of subgroups were referred to as terminal nodes. Diagnostic properties (including sensitivity, specificity, positive predictive value, negative predictive value, and likelihood ratio) were calculated. Cohen's kappa statistic was used to measure the level of agreement between LV systolic function predicted by echocardiography and that measured using the LVEF and the LVLS. The threshold for statistical significance was set to *P*<0.05. All statistical analyses were performed using GraphPad Prism software (version 8.0.0, GraphPad Software Inc., San Diego, CA, USA) and R version 4.0.5 (R Foundation for Statistical Computing, Vienna, Austria; www.r-project.org) through the RStudio interface Version 1.4.1106.

## Results

### Characteristics of the study patients

**Figure 1:**
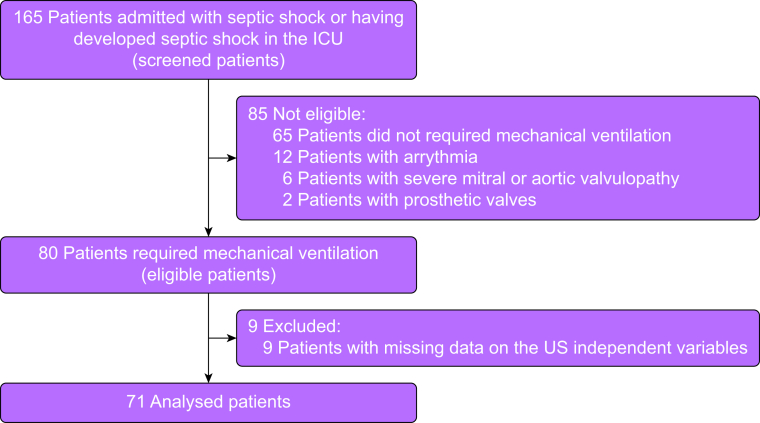
Study flowchart.

We included 71 ICU patients with septic shock receiving mechanical ventilation of the lungs (males: 61%; mean (sd) age: 61 [15]; mean [sd] Simplified Acute Physiology Score: 60 [19]) (Fig 1). Most of the cases were caused by community-acquired pneumonia (32%), ventilator-associated pneumonia (29%), or abdominal infections (23%). The median [IQR] arterial lactate and conventional troponin I concentrations were 4.6 [1.7–4.8] mmol L^−1^ and 7.6 [0.1–12.0] ng mL^−1^, respectively. All patients required noradrenaline, with a mean (sd) dose of 0.7 (0.7) μg kg^−1^ min^−1^. The median (IQR) heart rate and mean arterial pressure were 97 (79–111) beats min^−1^ and 73 (67–77) mm Hg, respectively (Table 1).

**Table 1:** Patient characteristics, biochemical and echocardiography data. Data are presented as median [inter-quartile], as mean (sd) or in numbers (%). ∗*n*=6 patients with missing data. CO, cardiac output; HR, heart rate; ICU, intensive care unit; LV-LWFS, left ventricular longitudinal wall fractional shortening; LVEF, left ventricular ejection fraction; LVLS, left ventricular longitudinal strain; MAP, mean arterial pressure; MAPSE, mitral annular plane systolic excursion; NA, noradrenaline; SAPS II, Simplified Acute Physiology Score; sd, standard deviation; TAPSE, tricuspid annular plane systolic excursion.

	All patients *n*=71
Clinical data	
Age, yr	61 (15)
Male sex, *n* (%)	43 (61)
SAPS II	60 (19)
Community-acquired pneumonia, *n* (%)	23 (32)
Ventilator-associated pneumonia, *n* (%)	21 (29)
Abdominal infections, *n* (%)	16 (23)
NA dose, μg kg^−1^ min^−1^	0.7 (0.7)
HR, beats min^−1^	97 [79–111]
MAP, mm Hg	73 [67–77]
CO, L min^−1^	6.2 (2.2)
CO <5.0 L min^−1^, *n* (%)	24 (34)
Biochemical data	
Lactate, mmol L^−1^	4.6 [1.7–4.8]
Troponin, ng ml^−1^	7.6 [0.1–12.0]
Echocardiographic data	
Left ventricular function	
LVEF, %	55 (13)
LVEF <50%, *n* (%)	22 (31)
LVLS, %	−14.1 (5.6)
LVLS greater than −17%, *n* (%)	52 (73)
Septal MAPSE, cm	1.02 (0.33)
Lateral MAPSE, cm	1.25 (0.37)
Septal S-wave velocity, cm s^−1^	9.8 (3.3)
Lateral S-wave velocity, cm s^−1^	12.0 (4.1)
LV-LWFS, %	11.8 (3.6)
Right ventricular function	
Lateral TAPSE, cm	1.84 (0.51)
Tricuspid S-wave velocity, cm s^−1^	13.9 (0.4)
Outcome	
ICU mortality, *n* (%)	41 (63%)∗

### Correlation between markers of LV systolic function

Intra- and inter-observer reliabilities of echocardiographic variables are shown in Supplementary Tables S3 and S4 and Supplementary Figs S1 and S2. The mean LVEF was 55% (13%) and 22 (31%) patients had an LVEF <50%. The mean LVLS was −14.1% (5.6%), and 52 (73%) patients had an LVLS greater than −17%. All patients with an LVEF <50% had an LVLS greater than −17% (Table 1).

The septal and lateral MAPSE were available in all patients, with mean values of 1.02 (0.33) cm and 1.25 (0.37) cm, respectively. The mean septal and lateral mitral S-wave velocities were 9.8 (3.3) cm s^−1^ (*n*=64) and 12.0 (4.1) cm s^−1^ (*n*=69), respectively. Lastly, the mean LV-LWFS was 11.8% (3.6%) (*n*=55) (Table 1).

The MAPSE, mitral S-wave, and LV-LWFS were significantly correlated with both LVEF and LVLS. Lateral MAPSE had the strongest correlation (r [95% confidence interval]) with LVEF (*r*=0.521 [0.321–0.676]; *P*<0.001), whereas septal MAPSE had the strongest correlation with LVLS (*r*=−0.638 [−0.470 to −0.761]; *P*<0.001) (Table 2).

**Table 2:** Correlations between markers of LV systolic function and LVEF or LVLS. CI, confidence interval; LV, left ventricular; LV-LWFS: left ventricular longitudinal wall fractional shortening, LVEF: left ventricular ejection fraction, LVLS: left ventricular longitudinal strain, MAPSE: mitral annular plane systolic excursion.

Variables	LVEF		LVLS	
	*r* [95% CI]	*P*	*r* [95% CI]	*P*
Septal MAPSE	0.458 [0.245–0.629]	<0.001	−0.638 [−0.470 to −0.761]	<0.001
Lateral MAPSE	0.521 [0.321–0.676]	<0.001	−0.491 [−0.285 to −0.654]	<0.001
Septal S-wave	0.463 [0.238–0.641]	<0.001	−0.378 [−0.138 to −0.576]	0.002
Lateral S-wave	0.361 [0.129–0.556]	<0.001	−0.243 [−0.001 to −0.459]	0.045
LV-LWFS	0.510 [0.283–0.683]	<0.001	−0.624 [−0.430 to −0.763]	<0.001

### An echocardiographic algorithm for predicting LV systolic function

**Figure 2:**
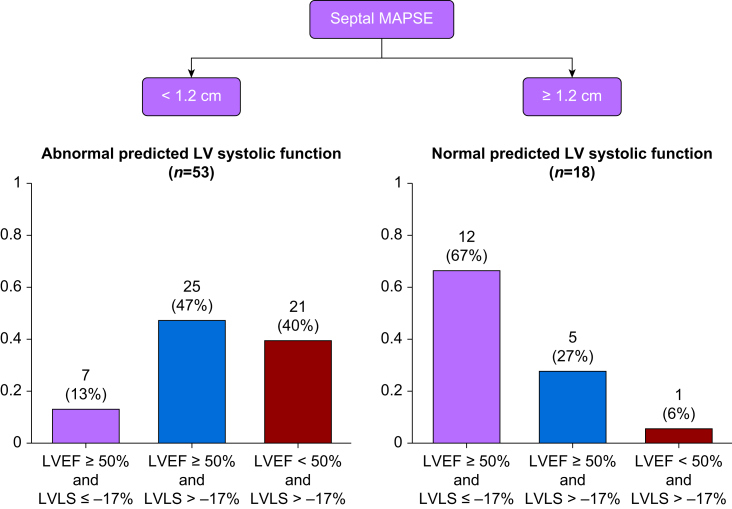
The proposed index for predicting LV systolic function. LV, left ventricular; LVEF, left ventricular ejection fraction; LVLS, left ventricular longitudinal strain; MAPSE, mitral annular plane systolic excursion.

Septal MAPSE (with a cut-off of 1.2 cm) was the best TTE predictor of LV systolic function as measured by LVEF and LVLS (Fig 2). The inclusion of other variables (such as the mitral S-wave and LV-LWFS) did not improve the algorithm's performance. Fifty-three patients (75%) had a septal MAPSE <1.2 cm, whereas 18 (25%) had a septal MAPSE ≥1.2 cm.

Among the patients with abnormal predicted LV systolic function (i.e. septal MAPSE <1.2 cm), 21 (40%) had both abnormal LVEF and abnormal LVLS. Interestingly, 25 patients (47%) had abnormal LVLS even though the LVEF was normal. Only seven patients (13%) had normal LVEF and normal LVLS (Fig 2).

In contrast, a large proportion of patients with normal predicted LV systolic function (i.e. septal MAPSE ≥1.2 cm) had both normal LVEF and normal LVLS (*n*=12, 67%). Only five patients (27%) had normal LVEF with abnormal LVLS, whereas one patient (6%) had both abnormal LVEF and abnormal LVLS (Fig 2).

The sensitivity, specificity, and positive and negative predictive values of the algorithm for predicting LV systolic function were 0.88, 0.63, 0.87, and 0.67, respectively (Table 3). The algorithm performance to predict an LVEF <50% or an LVLS greater than −17% are given in Supplementary Table S5. Cohen's kappa for the agreement between the algorithm and LV systolic dysfunction (defined as an LVEF <50%, an LVLS greater than −17%, or both) was 0.525 (0.299–0.751), which corresponds to moderate agreement. Performances of the other echocardiography variables to predict LV systolic dysfunction are provided in Supplementary Table S6.

**Table 3:** Accuracy of septal MAPSE for predicting LV systolic dysfunction. CI, confidence interval; LV, left ventricular; LVEF, left ventricular ejection fraction; LVLS, left ventricular longitudinal strain; MAPSE, mitral annular plane systolic excursion.

Variables	Impaired LV systolic function (LVEF <50%, LVLS greater than −17%, or both)	Preserved LV systolic function (LVEF ≥50% and LVLS less than or equal to −17%)	Total
Abnormal predicted LV systolic function (septal MAPSE ≤1.2 cm), *n* (%)	46 (87)	7 (13)	53
Normal predicted LV systolic function (septal MAPSE >1.2 cm), *n* (%)	6 (33)	12 (67)	18
Sensitivity			0.88
Specificity			0.63
Positive likelihood ratio			2.40
Negative likelihood ratio			0.18
Positive predictive value			0.87
Negative predictive value			0.67
Kappa agreement [95% CI]			0.525 [0.299–0.751]

## Discussion

We found that 31% and 73% patients with septic shock had LV systolic dysfunction, as assessed, respectively, by LVEF and LVLS. These prevalence valuesare consistent with previous studies.^24^^,^^25^ Because LVLS is an early, sensitive marker of systolic dysfunction, it might be a useful tool for helping clinicians to diagnose SCM. However, accurate measurements of strain (such as with GLS) require time (including off-line analysis), training, and expertise, which limits their routine use. Therefore, we proposed a simple index for predicting LV systolic function (including both LVEF and LVLS) in patients with septic shock.

We found that septal MAPSE was the best TTE variable for predicting LV systolic function. Septal MAPSE ≥1.2 cm predicted normal LVEF in 17/18 patients, but was not able to accurately detect altered LVLS. Septal MAPSE <1.2 cm almost always predicted LV systolic dysfunction with altered LVLS, while normal LVEF was maintained in almost half of the patients. Using MAPSE has several advantages. Firstly, MAPSE only requires a simple M-mode measurement, which can be obtained on all ultrasound systems including handheld devices. Secondly, MAPSE is easy to record—even in patients with a poor acoustic window (e.g. obese patients)—because the atrioventricular plane is strongly echogenic.^21^^,^^26^ In contrast, accurate measurements of LVEF or LVLS are dependent on the image resolution, in order to limit geometric and endocardial border assumptions. In a study of patients with sepsis, Johansson Blixt and colleagues^14^ demonstrated that MAPSE was easy to measure. In contrast, Boissier and colleagues^25^ demonstrated that even with trained operators, GLS was poorly feasible (in only 44% of cases) in the context of septic shock. Thirdly, bedside MAPSE measurement is highly reproducible (even for inexperienced operators) and requires minimal training.^27^, ^28^, ^29^, ^30^

MAPSE, S-wave velocity, LV-LWFS, LVEF, and LVLS analyse different mechanistic aspects of LV systolic function. For instance, LVEF is the sum of the contraction of circumferential, longitudinal, and oblique myocardial fibres, and is associated with a cardiac twist. Taken individually, these variables might not accurately reflect the true complexity of LV contractility. In physiological terms, myocardial contractility is related to the extent of shortening, the shortening velocity, and the contraction strength. Although ultrasound techniques cannot assess the contraction strength, MAPSE is a reliable index of longitudinal shortening. However, MAPSE and LV-LWFS are weakly related to LVEF, because only 60% of the LV ejection results from longitudinal contraction *per se*.^31^ It has been demonstrated that untwisting and decreased longitudinal contraction precede a reduction in LVEF in many heart diseases, such as hypertrophic cardiomyopathy.^28^ As long as circumferential contraction compensates for the decrease in longitudinal contraction, LVEF remains normal. These considerations might explain why (i) we and others found only a low to moderate positive correlation between LVEF and both septal and lateral MAPSE (0.458 and 0.521, respectively) and (ii) septal MAPSE <1.2 cm predicted an abnormal LVEF with a good sensitivity and negative predictive value.^13^ As expected, our index for predicting LVLS was more accurate than LVEF. Indeed, MAPSE and LVLS are well correlated.^13^^,^^14^

Neither LVEF nor mitral S-wave velocity are valid indicators of mortality in patients with septic shock.^8^^,^^32^ In contrast, GLS appears to be predictive of mortality in this population.^8^ Only two studies have demonstrated MAPSE to be an independent predictor of mortality in patients with septic shock.^29^^,^^33^ These findings suggest that septal MAPSE could be used for the easy and early detection of LV systolic dysfunction.

Our study had several limitations, most of which concerned the stratification algorithm. Firstly, we did not include all surrogates of LV systolic function (e.g. the maximum rate of change of pressure during the LV isovolumic contraction period [dP/dt] and the myocardial performance index). Moreover, we only measured septal and lateral MAPSE from the four-apical chamber view, regardless of the anterior and inferior walls (from the two-apical chamber view) and the anteroseptal and inferolateral walls (from the apical long axis view).^34^ Secondly, MAPSE may not reflect the LV systolic function in the presence of apical hypokinesia/dyskinesia (e.g. Takotsubo cardiomyopathy), since it focuses on the mitral annulus motion. Thirdly, we only reported the LVLS but this is in line with the majority of previous studies that included patients with septic shock, which also did not report the global circumferential and radial strains. GLS was usually measured in a 16- or 18-segment model as the mean segmental value from three apical imaging planes.^35^ The main limitation of this model is its feasibility because of poor echogenicity in many ICU patients. For this reason, we recorded the longitudinal strain from the four-apical chamber view, which mainly assesses the inferolateral and anteroseptal walls. However, Bazalgette and colleagues^36^ demonstrated a good level of agreement between GLS and the longitudinal strain from the four-apical chamber view. Fourthly, the M-mode technique is dependent on the insonation angle during image acquisition, which may lead to inaccurate measurement. Thus, an inappropriate angle tends to overestimate the mitral annular displacement and, therefore, the MAPSE. Conversely, LVLS is angle independent, as the speckle tracking occurs in the direction of the motion. Finally, we only included patients with adequate TTE image quality, in order to measure Doppler and strain variables in the whole study population. This should not have affected the measurement of septal MAPSE and the relevance of this index for predicting LV systolic function.

## Conclusion

Septal MAPSE with a cut-off of 1.2 cm was the best TTE variable for predicting LV systolic function in patients with septic shock. Since MAPSE corresponds to longitudinal LV shortening, it reflects LVLS more faithfully than LVEF. Hence, septal MAPSE might be a simple bedside tool for helping clinicians with the early detection of LV systolic dysfunction in a septic patient with conserved LVEF, especially when strain measurements are not available or feasible (e.g. in cases with poor echogenicity).

## Authors’ contributions

Designed the study: CB, YZ, JM, MS.

Collected the data: PM, JM, MS.

Analysed the data: CB, YZ, AM, MD.

Wrote the manuscript: CB, YZ, MS.

Critically reviewed the manuscript: PM, CT, JM.

Approved the final version of the manuscript: all authors.

Have agreed to be accountable for all aspects of the work by ensuring that questions related to the accuracy or integrity of any part of the work are appropriately investigated and resolved: all authors.

## Declarations of interest

The authors declare that they have no conflicts of interest.

## Data availability statement

The raw data supporting the conclusions of this article will be made available by the authors, without undue reservation.
