# Supplementary material for: Mitral annular plane systolic excursion for assessing left ventricular systolic dysfunction in patients with septic shock
Source: BJA Open. 2023 Aug 12;7:100220. doi: 10.1016/j.bjao.2023.100220 (PMC10457489; doi:10.1016/j.bjao.2023.100220)
Supplement: Multimedia component 1 [file mmc1.docx]

**Supplementary Material – Figure S1:**

Inter-observer agreements for left ventricular longitudinal strain (LVLS, in %), left ventricular ejection fraction (LVEF, in %), and septal mitral annular plane systolic excursion (MAPSE, in cm). A, C, E: Linear correlation plot for LVLS, LVEF, and septal MAPSE, respectively. B, D, F: Bland Altman plot for LVLS, LVEF, and septal MAPSE, respectively.
