# Supplementary material for: Mitral annular plane systolic excursion for assessing left ventricular systolic dysfunction in patients with septic shock
Source: BJA Open. 2023 Aug 12;7:100220. doi: 10.1016/j.bjao.2023.100220 (PMC10457489; doi:10.1016/j.bjao.2023.100220)
Supplement: Multimedia component 3 [file mmc3.docx]

**Supplementary Material - Table S1**

The Preferred Reporting Items for Critical care Echocardiography Studies (PRICES) checklist.

|  | **Checklist items** | **LV systolic function** | |
| --- | --- | --- | --- |
|  |  | **Collected** | **Reported** |
| **A1** | **Research vs clinical study** |  |  |
|  | - Research study | NA | Yes |
|  | - Clinical study | NA | No |
| **A2** | **Study information** |  |  |
|  | - Specific study type | NA | Yes |
|  | - State study design | NA | Yes |
|  | - Report sample size | NA | No |
| **A3** | **Patient information** |  |  |
|  | - Age | Yes | Yes |
|  | - Gender | Yes | Yes |
|  | - Height & weight (or BMI) | Yes | No |
|  | **Comorbidities** |  |  |
|  | - Ischaemic heart disease | No | No |
|  | - Atrial fibrillation | No | No |
|  | - Hypertension | No | No |
|  | - HFpEF | No | No |
|  | - HFrEF | No | No |
|  | - Pacemaker implant present | No | No |
|  | - COPD or pulmonary hypertension | No | No |
|  | - CKD or hemodialysis | No | No |
| **A4** | **Echocardiography information** |  |  |
|  | - Type of echo (TTE or TEE) | Yes | Yes |
|  | - Indicate if data collected at end-expiration | Yes | Yes |
|  | - No. of beats for averaging | Yes | Yes |
|  | - Report vendor of ultrasound machine | NA | Yes |
|  | - Indicate if airway pressure trace displayed on screen | NA | No |
| **A5** | **Clinical information at the time of echo** |  |  |
| **A5.1** | ***Ventilation*** |  |  |
|  | - Mode of ventilation | Yes | No |
|  | - Tidal volume | Yes | No |
|  | - Plateau pressure | Yes | No |
|  | - Positive end-expiratory pressure | Yes | No |
| **A5.2** | ***Hemodynamics*** |  |  |
|  | - Cardiac rhythm & heart rate | Yes | Yes |
|  | - Blood pressure | Yes | Yes |
|  | - Inotropes, vasopressors and doses | Yes | Yes |
| **A6** | **Reliability (for research study)** |  |  |
|  | - Feasibility of echo stated | Yes | Yes |
|  | - Intra-observer variability | No | No |
|  | - Extra-observer variability | No | No |
|  | - Indicate if observer blinded to treatment, if applicable | No | No |
| **A7** | **Statistics (for research study only)** |  |  |
|  | - Sample size calculation | No | No |
|  | - Indicate if statistician blinded to treatment / group | NA | NA |
|  | - Address confounders, if applicable | NA | NA |
|  | - Internal validation provided, if applicable | NA | NA |
| **B1** | **LV systolic function indices** |  |  |
|  | - LV ejection fraction | Yes | Yes |
|  | - Tissue Doppler S’ velocity | Yes | Yes |
|  | - Mitral annular systolic plane excursion | Yes | Yes |
|  | - LV strain or strain rate | Yes | Yes |
| **B2** | **LV size** |  |  |
|  | - LV end-diastolic diameter or volume | Yes | No |
| **B3** | **Other functional indices to aid interpretation** |  |  |
|  | - Cardiac output | Yes | Yes |
|  | - Stroke volume | Yes | No |
|  | - Any heart valve dysfunction | Yes | No |

BMI: body mass index, CKD: chronic kidney disease, COPD: chronic obstructive pulmonary disease, HFrEF: heart failure with reduced ejection fraction, HFpEF: heart failure with preserved ejection fraction, LV: left ventricular, TEE: transoesophageal echocardiography, TTE: transthoracic echocardiography.
