# Supplementary material for: Mitral annular plane systolic excursion for assessing left ventricular systolic dysfunction in patients with septic shock
Source: BJA Open. 2023 Aug 12;7:100220. doi: 10.1016/j.bjao.2023.100220 (PMC10457489; doi:10.1016/j.bjao.2023.100220)
Supplement: Multimedia component 4 [file mmc4.docx]

**Supplementary Material - Table S2**

Distribution of the missing variables

| **Parameters** | **n/n (%)** |
| --- | --- |
| LV-LWFS | 14/71 (19.2) |
| Septal MAPSE | 0/71 (0.0) |
| Lateral MAPSE | 0/71 (0.0) |
| Septal S-wave | 7/71 (9.9) |
| Lateral S-wave | 2/71 (2.8) |
| TOTAL | 333/355 (9.4) |

LVLS: left ventricular longitudinal strain, MAPSE: mitral annular plane systolic excursion.
