# Supplementary material for: Mitral annular plane systolic excursion for assessing left ventricular systolic dysfunction in patients with septic shock
Source: BJA Open. 2023 Aug 12;7:100220. doi: 10.1016/j.bjao.2023.100220 (PMC10457489; doi:10.1016/j.bjao.2023.100220)
Supplement: Multimedia component 5 [file mmc5.docx]

**Supplementary Material - Table S3:**

Inter-observer reliability of echocardiographic parameters (n=20)

| Correlation | Inter-observer agreement | | | | | | |
| --- | --- | --- | --- | --- | --- | --- | --- |
|  | Septal MAPSE | Lateral MAPSE | Septal S-wave | Lateral S-wave | LV-LWFS | LVEF | LVLS |
| r | 0.91  [0.79-0.97] | 0.85  [0.66-0.94] | 0.86  [0.67-0.94] | 0.88  [0.72-0.95] | 0.87  [0.74-0.93] | 0.89  [0.74-0.96] | 0.91  [0.77-0.96] |
| p | <0.0001 | <0.0001 | <0.0001 | <0.0001 | <0.0001 | <0.0001 | <0.0001 |
| R2 | 0.83 | 0.73 | 0.74 | 0.78 | 0.76 | 0.79 | 0.82 |
| Bias | 0.06  [-0.20-0.31] | 0.05  [-0.37-0.47] | <0.01  [-0.02-0.01] | <0.01  [-0.03-0.03] | 0.21  [-1.98-2.32] | 1.50  [-8.42-11.42] | 0.47  [-4.02-4.96] |
| SD of bias | 0.13 | 0.21 | 0.01 | 0.01 | 1.25 | 5.06 | 2.29 |

LV-LWFS: left ventricular longitudinal wall fractional shortening, LVEF: left ventricular ejection fraction, LVLS: left ventricular longitudinal strain, MAPSE: mitral annular plane systolic excursion.
