# Supplementary material for: Mitral annular plane systolic excursion for assessing left ventricular systolic dysfunction in patients with septic shock
Source: BJA Open. 2023 Aug 12;7:100220. doi: 10.1016/j.bjao.2023.100220 (PMC10457489; doi:10.1016/j.bjao.2023.100220)
Supplement: Multimedia component 6 [file mmc6.docx]

**Supplementary Material - Table S4:**

Intra-observer reliability of echocardiographic parameters (n=20)

| Correlation | Intra-observer agreement | | | | | | |
| --- | --- | --- | --- | --- | --- | --- | --- |
|  | Septal MAPSE | Lateral MAPSE | Septal S-wave | Lateral S-wave | LV-LWFS | LVEF | LVLS |
| r | 0.95  [0.89-0.98] | 0.89  [0.74-0.96] | 0.89  [0.73-0.95] | 0.90  [0.76-0.96] | 0.90  [0.77-0.90] | 0.85  [0.66-0.94] | 0.92  [0.82-0.97] |
| p | <0.0001 | <0.0001 | <0.0001 | <0.0001 | <0.0001 | <0.0001 | <0.0001 |
| R2 | 0.91 | 0.79 | 0.79 | 0.81 | 0.81 | 0.73 | 0.86 |
| Bias | 0.05  [-0.14-0.23] | 0.02  [-0.34-0.39] | -0.01  [-0.02-0.01] | 0.05  [-0.30-0.40] | 0.19  [-1.68-1.99] | 1.55  [-9.71-12.81] | -0.15  [-3.42-3.11] |
| SD of bias | 0.10 | 0.19 | <0.01 | 0.18 | 0.98 | 5.74 | 1.67 |

LV-LWFS: left ventricular longitudinal wall fractional shortening, LVEF: left ventricular ejection fraction, LVLS: left ventricular longitudinal strain, MAPSE: mitral annular plane systolic excursion.
