# Supplementary material for: Mitral annular plane systolic excursion for assessing left ventricular systolic dysfunction in patients with septic shock
Source: BJA Open. 2023 Aug 12;7:100220. doi: 10.1016/j.bjao.2023.100220 (PMC10457489; doi:10.1016/j.bjao.2023.100220)
Supplement: Multimedia component 7 [file mmc7.docx]

**Supplementary Material - Table S5**

Accuracy of septal MAPSE for predicting LVEF and LVLS.

| **Variables** | **LVEF <50%** | **LVEF ≥50%** | **Total** | **LVLS >-17%*** | **LVLS ≤-17%*** | **Total** |
| --- | --- | --- | --- | --- | --- | --- |
| Abnormal predicted LV systolic function (septal MAPSE ≤1.2cm) | 21 (40%) | 32 (60%) | 53 | 46 (87%) | 7 (13%) | 53 |
| Normal predicted LV systolic function (septal MAPSE >1.2cm) | 1 (6%) | 17 (94%) | 18 | 6 (33%) | 12 (67%) | 18 |
| Sensitivity |  |  | 0.95 |  |  | 0.88 |
| Specificity |  |  | 0.35 |  |  | 0.63 |
| Positive likelihood ratio |  |  | 1.46 |  |  | 2.40 |
| Negative likelihood ratio |  |  | 0.13 |  |  | 0.18 |
| Positive predictive value |  |  | 0.40 |  |  | 0.87 |
| Negative predictive value |  |  | 0.94 |  |  | 0.67 |
| Kappa agreement  [95%CI] |  |  | 0.217  [0.081-0.353] |  |  | 0.525  [0.299-0.751] |

* Because all patients with an impaired LVEF had also an impaired LVLS, the performance of the algorithm for predicting a LVLS >-17% corresponds to the performance of the algorithm for predicting LV systolic dysfunction.

LV: left ventricular, LVEF: left ventricular ejection fraction, LVLS: left ventricular longitudinal strain, MAPSE: mitral annular plane systolic excursion.
