# Supplementary material for: Mitral annular plane systolic excursion for assessing left ventricular systolic dysfunction in patients with septic shock
Source: BJA Open. 2023 Aug 12;7:100220. doi: 10.1016/j.bjao.2023.100220 (PMC10457489; doi:10.1016/j.bjao.2023.100220)
Supplement: Multimedia component 8 [file mmc8.docx]

**Supplementary Material - Table S6**

Performance of the other echocardiographic parameters for predicting LV systolic dysfunction (LVEF <50% and/or LVLS >-17%).

|  | **Lateral MAPSE** | **Septal S-wave** | **Lateral S-wave** | **LV-LWFS** |
| --- | --- | --- | --- | --- |
| Optimal cut-off value | 1.2 cm | 8.0 cm.s^-1^ | 8.0 cm.s^-1^ | 14.0% |
| Sensitivity | 0.63 | 0.48 | 0.33 | 0.98 |
| Specificity | 0.95 | 0.89 | 0.89 | 0.56 |
| Positive predictive value | 0.97 | 0.92 | 0.89 | 0.85 |
| Negative predictive value | 0.49 | 0.38 | 0.33 | 0.90 |

LV-LWFS: left ventricular longitudinal wall fractional shortening, LVEF: left ventricular ejection fraction, MAPSE: mitral annular plane systolic excursion.
